# 427. Trends in Hospital-onset *Escherichia coli* Infections Resistant to Fluoroquinolones and Extended-spectrum Cephalosporins, 2012-2020

**DOI:** 10.1093/ofid/ofac492.502

**Published:** 2022-12-15

**Authors:** Nicole Hood, Hannah Wolford, Babatunde Olubajo, Ashley Rose, James Baggs, Sujan Reddy

**Affiliations:** Emory University; CDC, Atlanta, Georgia; CDC, Atlanta, Georgia; CDC, Atlanta, Georgia; CDC, Atlanta, Georgia; CDC, Atlanta, Georgia

## Abstract

**Background:**

Inpatient and outpatient fluoroquinolone (FQ) use has decreased substantially in the last decade; however, the impact of this trend on FQ resistance is less clear. During this period, extended-spectrum β-lactamase (ESBL) *Escherichia coli* infections have increased. We examined hospital-onset (HO) *E. coli* clinical culture trends and resistance patterns among hospitalized adult patients in the United States.

**Methods:**

We measured the incidence of positive clinical cultures from inpatient encounters in a cohort of hospitals submitting data to the Premier Healthcare Database from 2012-2020. We included *E. coli* cultures with susceptibility testing to FQs or extended-spectrum cephalosporins (ESC). ESC resistance was defined as non-susceptibility to cefotaxime, ceftriaxone, ceftazidime, or cefepime and FQ resistance as resistance to ciprofloxacin, levofloxacin, or moxifloxacin. Co-resistance was defined as both ESC and FQ resistance in the same *E. coli* isolate. Isolates were classified as FQ resistant only (and not ESC), ESC resistant only (and not FQ), co-resistant, or neither FQ nor ESC resistant.

**Results:**

The overall proportion of HO *E. coli* with any FQ resistance decreased 12.3% (from 36.7% in 2012 to 32.2% in 2020). The proportion of HO *E. coli* with only FQ resistance decreased by 39.8% (from 27.9% in 2012 to 16.8% in 2020); the proportion with only ESC resistance increased by 112.5% (from 2.4% to 5.1%), and co-resistance increased by 75.0% (from 8.8% to 15.4%) (Figure).

**Figure.**
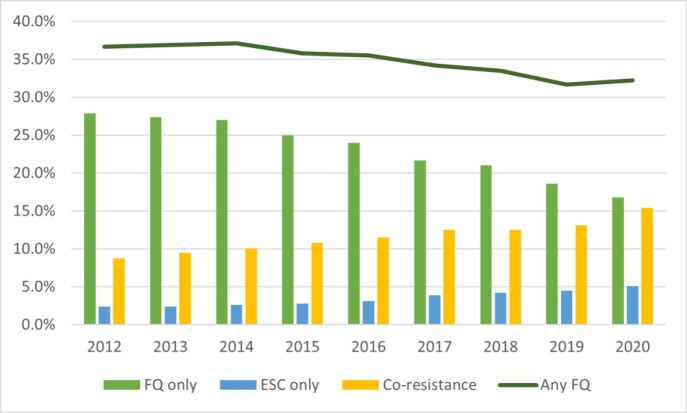
Proportion of hospital-onset E. coli infections with selected antibiotic resistance patterns, 2012-2020

**Conclusion:**

These results suggest that declining FQ use has been accompanied by declining FQ resistance in *E. coli*. Increases in FQ and ESC co-resistance may be due to factors including increases in the use of non-FQ antibiotics such as cephalosporins and increased transmission of *E. coli* strains such as ST131 in which co-resistance is prevalent. Antibiotic stewardship across multiple antibiotic classes combined with infection control measures are likely necessary to reduce multidrug resistant *E. coli* infections.

**Disclosures:**

**All Authors**: No reported disclosures.

